# Lysin and Lytic Phages Reduce Vibrio Counts in Live Feed and Fish Larvae

**DOI:** 10.3390/microorganisms12050904

**Published:** 2024-04-30

**Authors:** Jaime Romero, Sergueia Blas-Chumacero, Victoria Urzúa, Alejandro Villasante, Rafael Opazo, Felipe Gajardo, Claudio D. Miranda, Rodrigo Rojas

**Affiliations:** 1 Laboratorio de Biotecnología de Alimentos, Instituto de Nutrición y Tecnología de los Alimentos (INTA), Universidad de Chile, El Líbano 5524, Santiago 7830489, Chile; sergueia.blas@ug.uchile.cl (S.B.-C.); vrosariou@gmail.com (V.U.); alejandro.villasante@inta.uchile.cl (A.V.); ropazo@inta.uchile.cl (R.O.); fgajardoe@gmail.com (F.G.); 2 Laboratorio de Patobiología Acuática, Departamento de Acuicultura, Universidad Católica del Norte, Larrondo 1281, Coquimbo 1780000, Chile; cdmirand@ucn.cl (C.D.M.); rrojas@ucn.cl (R.R.)

**Keywords:** lysin, endolysin, Vibrio, lytic phage, rotifer, fish larvae, live feed

## Abstract

*Vibrio* species are naturally found in estuarine and marine ecosystems, but are also recognized as significant human enteropathogens, often linked to seafood-related illnesses. In aquaculture settings, Vibrio poses a substantial risk of infectious diseases, resulting in considerable stock losses and prompting the use of antimicrobials. However, this practice contributes to the proliferation of antimicrobial-resistant (AMR) bacteria and resistance genes. Our investigation aimed to explore the potential of biological agents such as bacteriophage CH20 and endolysin LysVPp1 in reducing Vibrio bacterial loads in both rotifer and fish larvae. LysVPp1’s lytic activity was assessed by measuring absorbance reduction against various pathogenic Vibrio strains. Phage CH20 exhibited a limited host range, affecting only *Vibrio alginolyticus* GV09, a highly pathogenic strain. Both CH20 and LysVPp1 were evaluated for their effectiveness in reducing Vibrio load in rotifers or fish larvae through short-setting bioassays. Our results demonstrated the significant lytic effect of endolysin LysVPp1 on strains of *Vibrio alginolyticus*, *Vibrio parahaemolyticus*, and *Vibrio splendidus*. Furthermore, we have showcased the feasibility of reducing the load of pathogenic Vibrio in live feed and fish larvae by using a non-antibiotic-based approach, such as lytic phage and endolysin LysVPp1, thus contributing to the progress of a sustainable aquaculture from a One Health perspective.

## 1. Introduction

Microbial colonization during fish development is influenced by both exposure and host selection [1]. Initially, bacteria attach to the surface of fish eggs and, subsequently, colonize various mucosal sites as newly hatched larvae consume their yolk sac. In marine fish, the act of drinking water for osmoregulation provides a potential source of microbes for gut colonization [2]. Furthermore, the first live feeds that larvae consume, such as the rotifers and Artemia commonly used in hatcheries, also play a role in shaping the gut microbiome [3]. This is very important when considering the bacterial load of the live feeds. Rotifer and Artemia cultures exhibited high levels of bacterial load, nearly 5 × 10^8^ Colony Formit Units (CFUs)/g [4]. The estimated bacterial load in live feed is 10^4^ bacteria per rotifer [5] and 5 × 10^4^ bacteria per Artemia [6].

Bacterial infections are among the most relevant problems associated with fish larvae mass mortalities. In fish larvae, the main route of entry for bacteria is through live feeds, after transition from endogenous to exogenous feeding. Rotifers (*Brachionus plicatilis*) are essential live prey in larval rearing of marine fish species; however, rotifers can be major carriers of bacteria [7]. Although, most of these bacteria are not pathogenic per se, detrimental effects on fish larvae can be caused by the increment of these bacteria in rotifers [8]. This is especially true since the bacteria associated with rotifer cultures have been linked to low survival and growth in fish larvae; however, when rotifers have been treated with chloramphenicol, furazolidone and sodium oxolinate, the survival rates of fish larvae are significantly improved [9,10]. Indeed, Gatesoupe [10], treated rotifers with antibiotics, after which observed greater survival in turbot larvae, suggesting bacteria associated with rotifers are harmful for the development of turbot larvae.

Among bacterial diseases, vibriosis (caused by bacteria of the genus Vibrio) has been reported to be the most prevalent disease in both marine fish and marine invertebrates hatcheries. The genus Vibrio has been found to be dominant in rotifers [11], and several Vibrio species have been reported to cause high mortality episodes in the culture of some fish species [12,13].

*Vibrio alginolyticus* is a pathogen for marine organisms, and is one of the most common bacteria found in marine hatchery water [14]. This microorganism is an opportunistic bacterium invading already-damaged tissues in fish [15]. There are several reports of significant mortalities in cultured gilthead seabream, *Sparus aurata*, especially during early life stages, caused by *V. alginolyticus* [16,17]. There is general consensus that *V. alginolyticus* enters fish larvae through live feed, especially rotifers, which serve as vehicles for introducing bacteria into the hatchery tanks [7,18,19].

Some treatments used for the disinfection of rotifers, such as the use of ozone and sanitizer agents, generate an inactivation of the rotifers [20]. On the contrary, Artemia is able to resist most of the chemical treatments and ultraviolet (UV) radiation [20,21,22,23,24]. Current techniques such as disinfection are not completely effective to achieve a complete bacteria-free marine hatchery environment. Furthermore, those treatments may lead to microbial imbalance, leaving an environmental niche wide open for the proliferation of opportunistic pathogens [8]. Hence, innovative and environmentally friendly solutions to control vibrio infections in marine fish larvae are required. A feasible option to do so is the use of phage therapy or phage-endolysin tools [25].

Bacteriophages have been proposed as antimicrobials against pathogenic bacteria in different fields, including aquaculture [26]. The literature shows examples of phage applications focusing in the control of pathogenic Vibrios, one the major problems in aquaculture [27,28,29]. Despite those promising reports on this topic, there is always associated risk in the release of phages to the environment, especially in aquaculture systems [30]. One of the main risks it is associated with is the proliferation of new virulent bacteria.

Endolysins (also called lysins) are proteins produced by bacteriophages in the late stages of infection. These enzymes degrade bacterial cell wall peptidoglycan (PG), finally killing the infected bacterial host. Bacteriophage research has improved our knowledge about endolysins and their role in the bacteriophage lytic cycle, as well as applications of endolysins as antimicrobials. Endolysins can be a promising antibacterial alternative to control bacterial infection in aquaculture. Their mechanism of action, based on hydrolysis of cell wall bonds, reduces the probability of resistance development, and they are considered safe for eukaryotic organisms, hence friendly to organisms used as live feed, such as rotifers [31], as well as implying no risk for consumers’ health. Based on wide evidence that supports its effectiveness both in vitro and in vivo evaluations, endolysins have been recognized as promising antibacterial agents [32]. However, the potential use in the aquaculture field has not been explored yet. In this study, we conducted an assessment of the effectiveness of a lytic phage and endolysin to reduce the vibrio counts in both rotifers population and fish larvae. Our investigation aimed to explore the potential use of these biological agents in mitigating vibrio bacterial loads, which pose a significant concern in the aquaculture industry due to their detrimental impact in health and development of fish larvae.

## 2. Materials and Methods

### 2.1. Vibrio Strains

Various *Vibrio* species were incorporated into this study, due to their capacity to induce diseases in multiple hosts, including fish, humans, and shellfish. Specifically, the selected species were *Vibrio alginolyticus*, *Vibrio parahaemolyticus*, and *Vibrio splendidus*. One strain of *Vibrio alginolyticus* (GV09) was included because it harbors antibiotic-resistance genes and was associated with high mortalities in red cusk eel larvae (*Genypterus chilensis*) [4]. One strain of *Vibrio parahaemolyticus* (PMC57.5) was selected due to its association with a clinical case linked to a shellfish consumption outbreak previously documented in Chile [33]. Three strains of *Vibrio splendidus* (VPAP16, VPAP18, and VPAP23) were included because they were previously identified as the most prevalent organisms during three separate incidents of widespread larval culture mortality in commercial hatcheries situated in northern Chile. These incidents involved larval cultures of the Chilean scallop *Argopecten purpuratus* [34].

### 2.2. Lytic Phage and Endolysin

The studied the endolysn LysVPp1 was described previously [35]. LysVPp1 was synthesized by GenScript. A synthetic gene was cloned into the *E. coli* expression vector pET-30, and the plasmids were transformed in BL21 (DE3) for protein expression. The *E. coli* strain was cultured overnight at 37 °C. The following day, the culture was diluted 1:100 in fresh medium and cultured with agitation at 160 rpm until reaching an OD600 of 0.6. Protein overexpression was induced with IPTG (0.1 mM) for 20 h. The culture was centrifuged at 5000× *g* for 6 min, and the pellets were resuspended in 9 mL of lysis buffer [36] and sonicated with 10 pulses of 30 s on and 30 s off at 60% amplitude. The samples were then centrifuged at 10,000× *g* for 20 min, and each supernatant was filtered through a 0.45 μm filter. The proteins were purified using a HisPur Ni-NTA purification kit (Thermo Scientific, Waltham, MA, USA) according to the manufacturer’s instructions. Protein concentration was measured using a Qubit.

The phage CH20 was isolated from mussel homogenate following the procedure described by [27], utilizing GV09 as the host strain. The phage CH20 was partially characterized, including determination of its host range, plaque efficiency, latency, microscopy analysis, nucleic acid, Direct genome restriction enzyme analysis (DGREA) and genome sequencing, as described previously [27,37].

### 2.3. Lytic Activity of Endolysin on Vibrio Strains

The lytic activity of endolysin LysVPp1 on vibrio strains was evaluated following the protocol outlined by Li [35]. The preparation of bacteria involved an overnight culture (15 mL), followed by centrifugation to obtain a pellet (5000× *g* for 6 min), resuspension in Phosphate-Buffered Saline (PBS; 10 mL), and subsequent centrifugation. The pellet was then washed twice with autoclaved distilled water. Prior to the assay, the bacteria were resuspended in 3 mL of reaction buffer (20 mM NaH_2_PO_4_ [pH 8.0] containing 0.1% Triton X-100). The lytic activity assay was performed using a spectrophotometer with a 1 mL cuvette, measuring optical density at 600 nm. The treatment groups received LysVPp1 concentrations of 1 mg/mL or 2 mg/mL, while the control group received a volume of elution buffer equivalent to that of the protein in the treatment group. Absorbance was measured every minute for 10 min.

### 2.4. Vibrio Reduction in Bioassay Using Rotifers

We employed the endolysin LysVPp1 and the phage CH20 to assess their efficacy in reducing the Vibrio load in the rotifer *Brachionus plicatilis*. Rotifer cultures were performed as described in [18]. Rotifers were filtered over a nylon mesh of 30 μm pore size and re-suspended in 1μm filtered seawater. A volume of a Vibrio strain GV09 culture was added to rotifer cultures, reaching 5 × 10^6^ CFU per mL. After 15 min of incubation at 22 °C, the mixture was aliquoted into portions of 9 mL onto sterile plates, including approximately 350 rotifers per well. For the control group, 1 mL of seawater was added. For the endolysin treatment, 1 mL of LysVPp1 was added to achieve a final concentration of 1 mg per mL. For the phage treatment, 1 mL of CH20 was added to achieve a multiplicity of infection (MOI) of 100. All assays were performed in triplicate. After 20 min, rotifers from each treatment were filtered using a 30 μm mesh, collecting 5 mL of medium. The rotifers were then washed and re-suspended in seawater, as described in [4]. The load of pathogenic Vibrio in both the rotifers and the water was measured by bacterial counts on Thiosulfate–Citrate–Bile Salts–Sucrose (TCBS) agar.

### 2.5. Vibrio Reduction in Bioassay Using Fish Larvae

Zebrafish larval cultures (*Danio reiro*) were conducted as described by [38] (Opazo 2019). Vibrio strain GV09 culture was added to the fish larval cultures, achieving a concentration of 5 × 10^6^ CFU per mL. After 15 min of incubation at 22 °C, the mixture was aliquoted into portions of 9 mL onto sterile plates, 6 fish larvae per well. For the control group, 1 mL of E3 medium was added. For the endolysin treatment, 1 mL of LysVPp1 was added to achieve a final concentration of 1 mg per mL. For the phage treatment, 1 mL of CH20 was added to achieve a multiplicity of infection (MOI) of 100. All assays were performed in triplicate. After 20 min, fish larvae and medium were collected as described in Section 2.4. The load of pathogenic Vibrio in both the fish larvae and the water was measured by bacterial counts on TCBS agar. Animal procedures adhered to the recommendations outlined in the “Guide for the Care and Use of Laboratory Animals of the National Institutes of Health” and the animal ethics guide of University of Chile, Ethical code 20364-INTA-UCH.

## 3. Results

### 3.1. Lytic Activity of Lysin against Vibrio Strains

**Figure 1:**
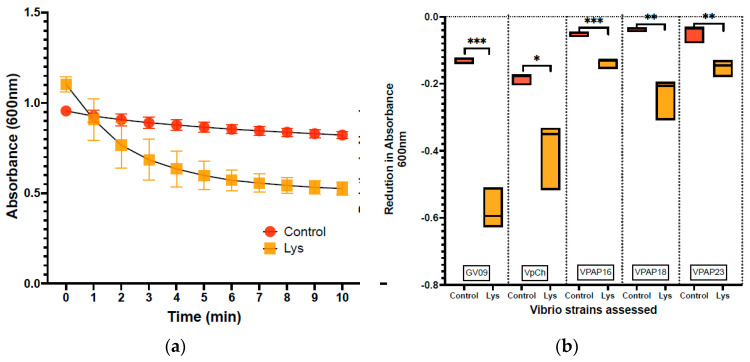
Lytic activity of lysin (LysVPp1) against Vibrio strains. (**a**): The lytic activity of Lysin on strain GV09 is depicted, with a focus on monitoring the absorbance at 600 nm throughout the assay period (10 min). (**b**): Boxplot showing the reduction in absorbance observed from the beginning (0 min) to the end of the assay (10 min) for both the control group and the Vibrio strains (GV09; VpPMC57.5; VPAP16; VPAP18; VPAP23) treated with LysVPp1.

The results of the lytic assay, which relied on the reduction in absorbance, revealed a notable decrease in absorbance values over time, indicating the lysis of Vibrio cells by lysin. For instance, the reduction in absorbance for strain GV09 is depicted in Figure 1a, where a fast lytic activity was observed during the first 5 min of the assay. Additionally, the extent of reduction varied across different Vibrio strains tested, as illustrated in Figure 1b, which demonstrates the reduction in absorbance for each evaluated Vibrio strain. The effect of lysin (LysVPp1) was particularly remarkable on strains of *Vibrio alginolyticus* (GV09) and *Vibrio parahaemolyticus* (PMC57.5). In the case of *V. alginolyticus* (GV09), the reduction in absorbance was −0.4433 ± 0.0353, while for *V. parahaemolyticus* (PMC57.5), it was −0.2150 ± 0.0594. Moreover, both strains exhibited a statistically significant reduction in absorbance compared to the control group, as determined by *t*-test analysis (*p* = 0.0002 and *p* = 0.0024, respectively). The impact on *V. splendidus* strains (VPAP16, VPAP18, and VPAP23) was less pronounced, with an average reduction in absorbance of −0.1292. Nevertheless, the reduction was still statistically significant, as determined by Welch’s test (*p* = 0.0026, *p* = 0.0310, and *p* = 0.0081, respectively), indicating a measurable effect of the lysin treatment on these strains (Table 1). It is interesting to note that an increase in the enzyme dosage can improve the results in this analysis. For example, the use of 2 mg/mL can achieve significant reductions on the order of −0.3 in absorbance in the VPAP23 strain (Figure S1).

### 3.2. Phage CH20 against Vibrio Strain GV09

**Figure 2:**
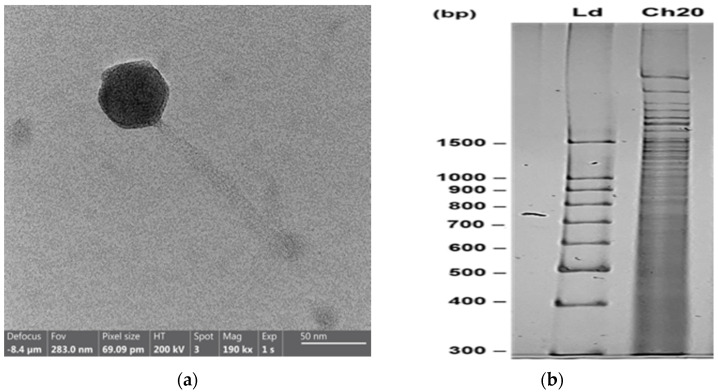
Properties of phage CH20. (**a**): Electron microscopy. (**b**): PAGE showing the genome restriction enzyme analysis using *Taq*I. Ld: Ladder 100 bp; Ch20: phage genome digested.

Given that strain GV09 exhibited the highest susceptibility to the endolysin LysVPp1, and is characterized as a multi-resistant strain to antibiotics, which has been linked to mortalities in hatcheries where live food is implicated, our investigation focused on identifying a lytic phage capable of targeting GV09 (Table 1). The partial characterization of phage CH20 is detailed in the Supplementary Materials (Table S1; a one-step curve and genome sequence is shown in Figure S2). Figure 2a depicts the siphoviral morphology of phage CH20. Figure 2b illustrates the pattern obtained in polyacrylamide gel electrophoresis (PAGE) after digestion of the genome DNA using *Taq*I.

### 3.3. Reduction in Vibrio Load in Rotifers

**Figure 3:**
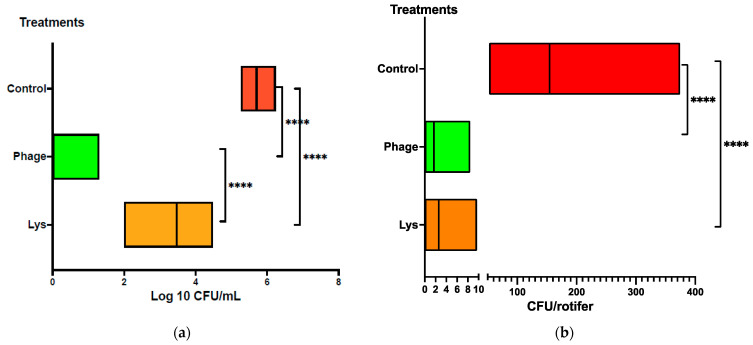
Reduction in Vibrio counts after treatments. (**a**) Vibrio counts in rotifer culture media (seawater, Log10) and (**b**) Vibrio counts in rotifers. **** indicate statistical significance.

In the rotifer bioassay, we aimed to evaluate the effectiveness of either LysVPp1 or CH20 in decreasing the Vibrio strain GV09 counts. The assay is tailored to last just 20 min, aligning with the swift requirements of potential applications in an aquaculture hatchery. The objective was to treat the rotifer swiftly to reduce Vibrio bacteria, and thus enhancing its microbiological quality as a live food source for fish larvae. In order to better visualize the treatment effect, the reduction in Vibrio was measured both in the water and in the rotifers. In seawater, the reduction in vibrio counts (GV09 counts) was notably significant following the phage CH20 treatment, surpassing 5 logs reduction, as depicted in Figure 3a. Similarly, with the endolysin LysVPp1, a substantial reduction was observed, amounting for a 2-log decrease. Furthermore, both treatments demonstrated statistically significant reductions in viable GV09 counts compared to the control group, as determined by *t*-test analyses (*p* < 0.0001 and *p* = 0.0079, respectively). Interestingly, phage CH20 caused a greater effect than endolysin in reducing vibrio counts, as determined by *t*-test analysis (*p* < 0.0001).

In rotifers, Vibrio load reached an average of 200 CFU per rotifer in the control group. Both treatments, LysVPp1 and CH20, showed a reduction close to 95% in Vibrio counts per rotifer, as depicted in Figure 3b. Moreover, treatments exhibited a statistically significant reduction in Vibrio counts compared to the control group, as determined by *t*-test analyses (*p* = 0.0002 for each).

### 3.4. Reduction in Vibrio Load in Fish Larvae

**Figure 4:**
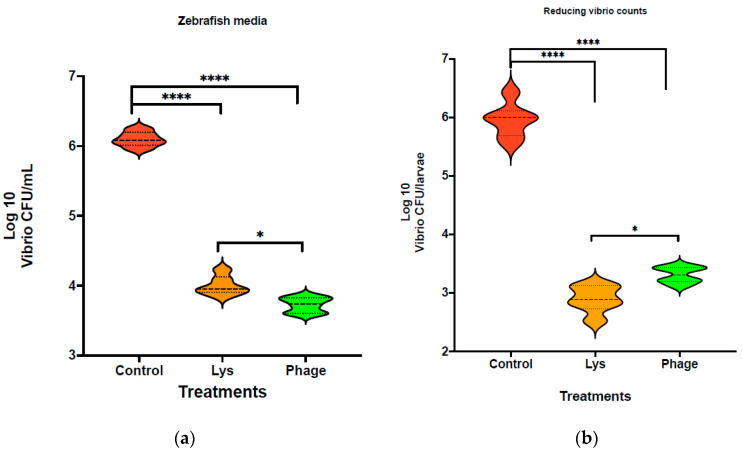
Reduction in Vibrio counts in zebrafish larvae after treatments. (**a**) Vibrio counts (Log10) in medium and (**b**) Vibrio counts in fish larvae.

Using the larvae bioassay, we aim to determine whether LysVPp1 or CH20 decrease Vibrio strain GV09 counts after 20 min of treatments. A reduction in Vibrio was measured in both the medium and larvae in order to better visualize the treatment effect. Following LysVPp1 treatment, the vibrio count (GV09 count) decreased significantly, by at least 2 logs in the medium, as shown in Figure 4a. With the phage CH20, a 2-log decrease in the medium was also observed. Furthermore, both treatments demonstrated statistically significant reductions in viable GV09 counts compared to the control group, as determined by *t*-test analysis (*p* < 0.0001). Notably, the comparison of treatments showed that phage CH20 was notably more effective at reducing vibrio counts compared to endolysin, as determined by *t*-test analysis (*p* < 0.0031).

In larvae, the Vibrio load in the control group averaged 10^6^ CFU per larva. Following treatment with phage CH20, a reduction of 2.6 logs was observed, resulting in a decrease to less than 10^4^ CFU per larva. Remarkably, the LysVPp1 treatment achieved a superior reduction in Vibrio load, exceeding 3 logs, which equates to less than 10^3^ CFU per larva, as illustrated in Figure 3b. Moreover, both treatments exhibited a statistically significant reduction in Vibrio load compared to the control group, as determined by *t*-test analyses (*p* < 0.0001 for each). It is noteworthy that the LysVPp1 treatment outperformed the phages, achieving an over 1-log reduction in vibrio load, and this difference was statistically significant, as determined by *t*-test analysis (*p* < 0.0033).

## 4. Discussion

Bacteriophages have been proposed as antimicrobials against pathogenic bacteria in different fields, including aquaculture [26]. The literature shows examples of phage applications focusing on the control of pathogenic Vibrios, one the major problems in aquaculture [27,28,29,39,40]. Following this research trend, in this study, a lytic phage was isolated to control the GV09 strain of antibiotic-resistant Vibrios. Phage CH20 has a siphoviral morphology. These phages typically have an icosahedral head and a long, flexible, non-contractile tail [41]. The tail is often longer than the head, and may have fibers or other appendages [42]. Among tailed phages, those with siphoviral morphology are the most abundant, accounting for 60% of the documented observations [43]. CH20 has double-stranded DNA genome, as shown in the genome digestion assay [43]. Genome sequencing indicates that CH20 genome (80 Kb) is similar to Vibrio phage vB_VorS-PVo5 (99,2% identity; 43% coverage) isolate found in Antofagasta, Chile (Genbank KT345706.1; Figure S3), belonging to the Ermolyevavirinae subfamily.

Our investigation revealed that phage CH20 exhibited a relatively short latent period of approximately 10 min, along with a burst size of 42 viral particles per infected cell. Similarly, phage vB_ValP_IME271 displayed a comparable burst size of 40 pfu/cell, but showed an extended latent period of 90 min [44]. In another example, phage P23 demonstrated a burst size of around 24 pfu/cell, coupled with a latent period of 30 min [45]. Contrasting with these findings, prior studies on Vibriophages documented larger burst sizes but longer latent periods. Additionally, phage BUCT549 exhibited a latent period of approximately 30–40 min and an average burst size of 141 pfu/cell [46]. In another example, phage V-YDF132 displayed a latent period of 20 min and a burst size of 298 pfu/cell [47]. Conversely, vB_VcaS_HC had a notably longer latent period of 1.5 h, but showcased a larger burst size of 172 pfu per infected cell [48].

The host range of CH20 was limited, in contrast to other Vibrio phages such as PcB-1G, which exhibited a broad range of lytic activity. It was found that 61% (33 out of 54) of *V. harveyi* isolates could be inhibited by the phage PcB-1G [39]. CH20 showed a similar host spectrum to the phage VPp1, which lysed only 3 out of 12 *V. parahaemolyticus* strains [35]. This narrow specificity is an advantage, in the sense that phages will not disrupt host microbiota; however, it demands that each phage be screened against each bacterial target to determine susceptibility prior to treatment [49]. Phage cocktails help mitigate the development of phage-resistant variants in bacteria and broaden the spectrum of bacterial strains that can be targeted. However, phage cocktails require the use of multiple phages, which can complicate the formulation and administration of the treatment [50]. Moreover, the interaction between bacteriophages and their bacterial targets is dynamic, leading to the possibility of the bacteria developing resistance to the bacteriophages. Furthermore, there is always associated risk in the release of phages to the environment, especially in aquaculture systems [30]. One of the main risks is the selection pressure towards new virulent bacteria due to the risk of horizontal gene transfer between phages and bacteria [51]. In this regard, endolysins, when used as recombinant enzymes, overcome the restrictions associated with the transmission of genetic material, and may offer other advantages such as the absence of resistance generation, environmental friendliness, and biodegradability.

LysVPp1, derived from the phage VP1p1 of the Myoviridae family, is known to target *V. parahaemolyticus* strains [35]. It is characterized as a soluble lytic transglycosylase, similar to hen egg white lysozyme, with an approximate molecular weight of 30 kDa. Notably, LysVPp1 lacks a reported peptidoglycan binding domain. Additionally, the phage genome also contains annotations for noholins/antiholin [35].

The enzyme LysVPp1 demonstrated the ability to break down 9 out of 12 strains of *V. parahaemolyticus*, indicating a wider range of targets compared to its associated phage VPp1, which was only effective against 3 of the 12 *V. parahaemolyticus* strains [35]. Likewise, the endolysin LysVpKK5 demonstrated the most potent activity against the VPATCC-17802 strain, whereas the original VpKK5 phage failed to infect the reference strain (Lal 2016) [52]. So far, the lytic capacity of some Vibriophage endolysins has been evidenced on sensitized cells of *V. mimicus*, *V. anguillarum*, *V. harveyi*, *V. alginolyticus* and *V. parahaemolyticus* [53,54,55].

In our study, the reduction in absorbance was mainly observed in the first 5 min of the assay (Figure 1a). This fast drop in absorbance has also been observed in other endolysin assays. Lysqdvp001 reduced turbidity of host bacteria by 0.6 log upon 5 min of incubation (Wang 2016) [56]. The structure of Lysqdvp001 comprises two domains: a PG_binding_1 (PF01471) domain and a cysteine, histidine-dependent amidohydrolases/peptidases (CHAP) domain (PF05257) [56]. Other studies on LysVPp1, as reported by [35], demonstrated a reduction of 0.4 log after 5 min of incubation with *V. parahaemolyticus*, mirroring our findings. There are other reports on the action of endolysins on bacteria in in vitro assays; however, these assays reported the results as percentage reductions in absorbance, and typically as endpoint measurements [57].

Destabilizing the outer membrane is crucial to facilitate the access of endolysins to the cell wall when applied externally. Various studies have reported that membrane permeabilizers, such as chelators, cationic agents, and essential oils, enhance the sensitivity of Gram-negative bacteria to the bactericidal effects of endolysins, serving as adjuvants to potentiate or induce their action [58]. Consequently, the differences in activity observed across different strains in our study and previous reports are primarily attributed to variances in susceptibility to permeabilization treatment rather than disparities in their cell wall composition, which remains relatively consistent among Gram-negative bacteria [58,59,60]. In this context, Zermeño-Cervantes [61] evaluated the efficacy of KZ144 and LysPA26 endolysins against *V. parahaemolyticus* in natural seawater. The results revealed that both endolysins displayed lytic activity in seawater, although KZ144 exhibited a reduction compared to the control condition. Although not directly tested, the influence of freshwater or seawater on our results can be inferred. By comparing the reductions in Vibrio counts in the rotifer assay, conducted in seawater, with the bioassay in zebrafish larvae, conducted in low-salinity medium, it is evident that LysVPp1 was not significantly affected by the saline environment (2 log reduction in both conditions). Endolysins with lysozyme activity, such as LysVPp1, are regarded as promising antibacterial agents for controlling and combating pathogens and multidrug-resistant bacteria [62]. Lysozyme has been utilized as an active ingredient in microbeads and attached to the mouths of rotifers as a strategy to reduce the viability of *E. coli* [63]. This approach leverages the antibacterial properties of lysozyme to target harmful bacteria, by using rotifers as vectors for the lysozyme-containing microbeads [63,64].

Rotifers used as live feed host a wide array of microbiota. While these microorganisms are typically non-pathogenic to the feed organisms themselves, they can be passed on to larval predators and may cause harmful effects. The use of antibiotics appeared to be a straightforward solution to address these issues. However, their prophylactic administration led to several secondary effects, including an increase in the frequency of strains developing antibiotic resistance [4].

The significance of live feed, such as rotifers and Artemia, as vectors in disease transmission has been emphasized, due to the confirmed transfer of the nervous necrosis virus (NNV) to the larvae of the Senegalese sole [65]. Applications of disinfection procedures, such as the ones successfully used in Artemia hatching and enrichment, are lethal for rotifers [8]. Consequently, numerous studies have been undertaken to diminish the bacterial load in rotifers before feeding them to fish larvae. One study found that the bacterial load of the rotifers decreased by 90%, from 7.35 × 10^3^ to 0.79 × 10^3^ CFU per rotifer, representing a reduction of only 1 log after 30 min of UV exposure [24]. In another example, a 15 min treatment with hydrogen peroxide effectively reduced the counts of heterotrophic bacteria from 7.88 × 10^2^ to 8.50 × 10^1^ CFU per rotifer, almost one order of magnitude (1 log) [66]. Those reductions are limited compared to the phage or endolysin (LysVPp1) treatments, which reduce the bacterial load from 200 CFU to 3 CFU per rotifer; i.e., more than 1 log, in a short time assay (20 min). This underscores the potential of phage or endolysin treatments in efficiently reducing bacterial contamination in rotifer cultures for aquaculture purposes.

The use of endolysins in fish larvae is a relatively new and developing area of research, and studies are being conducted to evaluate their efficacy, safety, and viability in different aquaculture systems [50]. This approach is part of a broader effort to find alternatives to conventional antibiotics and to reduce the impact of bacterial diseases in aquaculture. The use of phages or phage therapy has been studied in larvae; however, the focus was from the perspective of treatment, that is, reducing mortality, rather than reducing the levels of the pathogen, to prevent disease conditions [50,67,68]. For example, in zebrafish larvae [68], the mortality rate in the group of larvae that was infected with *Vibrio anguillarum* and phage-treated was notably lower than in the group that was infected but not phage-treated, demonstrating the effectiveness of the phage treatment. In the case of shrimp larvae, the early application of phage mixture (at 6 h post-infection) was effective to avoid mortality (80% survival) [67]. The use of endolysins for the control of early shrimp disease (AHPND), caused by Vibrio, is still in the evaluation stage of in vitro trials on the different strains reported to be associated with this disease [61].

Reducing the number of pathogenic or opportunistic bacteria as a strategy to prevent disease is a critical approach in various fields, including aquaculture. This strategy focuses on controlling the bacterial population to a level where it is less likely to cause infections or disease outbreaks. In aquaculture, treatments on larvae become operationally complicated, especially if there is a large volume to treat. Therefore, the focus is on treating the sources of pathogenic bacteria. Among these, rotifers require gentle methods that do not damage their structure and allow for viability. There are some studies that have attempted to apply treatments to larvae to reduce the bacterial load. For instance, Vallés [69] investigated the disinfection efficacy of hydrogen peroxide on meagre larvae. Before treatment, larvae at 13 days post-hatching (dph) exhibited high levels of total heterotrophic bacteria (8 × 10^5^ CFU per larva). Post-application of hydrogen peroxide, these levels dropped significantly, to 3 × 10^4^ CFU per larva. In a similar vein, counts of Vibrionaceae markedly decreased from 3 × 10^3^ to 7 × 10^2^ CFU per larva as an outcome of the treatment. Remarkably, our study showed that the LysVPp1 treatment achieved a superior reduction in vibrio load, exceeding 3 logs, which translates to less than 10^3^ CFU per larva, while the phage CH20 reduced the count to 10^4^ CFU per larva. By focusing on reducing the number of harmful bacteria, it is possible to maintain a healthier and more balanced environment, which is crucial for preventing the onset of diseases. This strategy is especially important in closed systems like aquaculture, where the spread of pathogens can be rapid and devastating [50].

## 5. Conclusions

Our findings provide compelling evidence for the viability of reducing the burden of pathogenic Vibrio in live feed and fish larvae through the utilization of antibiotic alternative tools, specifically lytic phages and endolysin Lys. The efficient and rapid action of these treatments highlights their potential for application in hatcheries. This promising aspect underscores the importance of exploring and implementing such strategies as part of sustainable aquaculture practices. By mitigating the presence of pathogenic bacteria in crucial stages of fish development, these interventions not only contribute to enhanced health and welfare, but also support the long-term sustainability of aquaculture operations from a One Health perspective.

## Figures and Tables

**Table 1:** Lytic effect of LysVPp1 and phage CH20.

Vibrio Strain	LysVPp1 Lysis	CH20 Lysis
*V. alginolyticus* GV09	+	+
*V. parahaemolyticus* PMC 57.5	+	-
*V. splendidus* VPAP16	+	-
*V. splendidus* VPAP18	+	-
*V. splendidus* VPAP23	+	-

The lytic effect was denoted with symbols where a positive (+) indicates a positive lytic effect and a negative. ONLY THREE (-) signifies no lytic effect observed.

## Data Availability

Data are contained within the article and Supplementary Materials.

## Supplementary Materials

The following supporting information can be downloaded at: https://www.mdpi.com/article/10.3390/microorganisms12050904/s1, Figure S1: Lytic activity of lysin (Lys) against Vibrio strain VPAP23 using 1mg/mL/Lys-1) or 2 mg/mL (Lys-2); Figure S2: One step curve of the phage CH20 on Vibrio GV09; Figure S3. Tree generated by BLAST using the Fast Minimum Evolution method; Table S1. Phage CH20 partial characterization.
