# Supplementary material for: Lysin and Lytic Phages Reduce Vibrio Counts in Live Feed and Fish Larvae
Source: Microorganisms. 2024 Apr 30;12(5):904. doi: 10.3390/microorganisms12050904 (PMC11123823; doi:10.3390/microorganisms12050904)
Supplement: Supplementary file 1 [file microorganisms-12-00904-s001.zip › microorganisms-2920761-supplementary-R1.pdf]

Lysin and lytic phage reduce vibrio counts in live feed and fish larvae

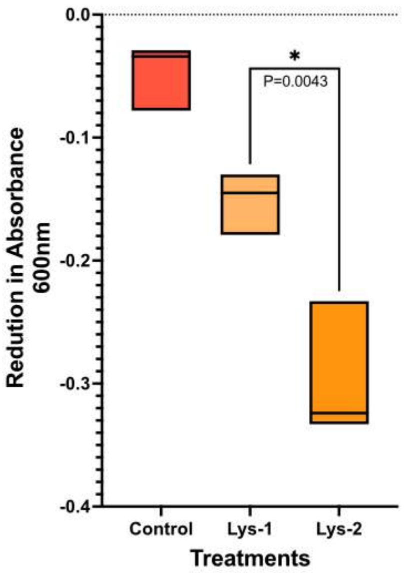

Figure S1. Lytic activity of Lysin (Lys) against Vibrio strain VPAP23 using 1mg/mL /Lys-1) or 2mg/mL (Lys-2)

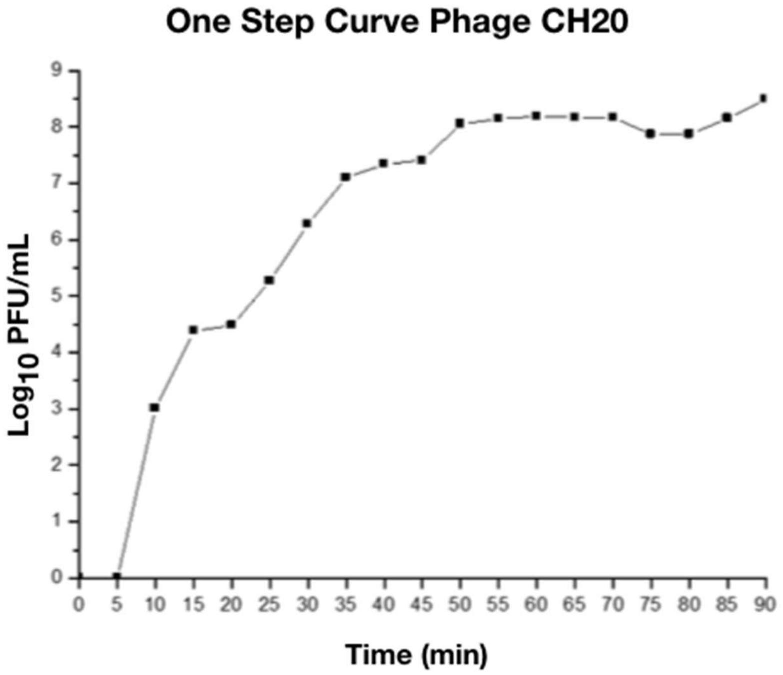

Figure S2. One step curve of phage CH20.

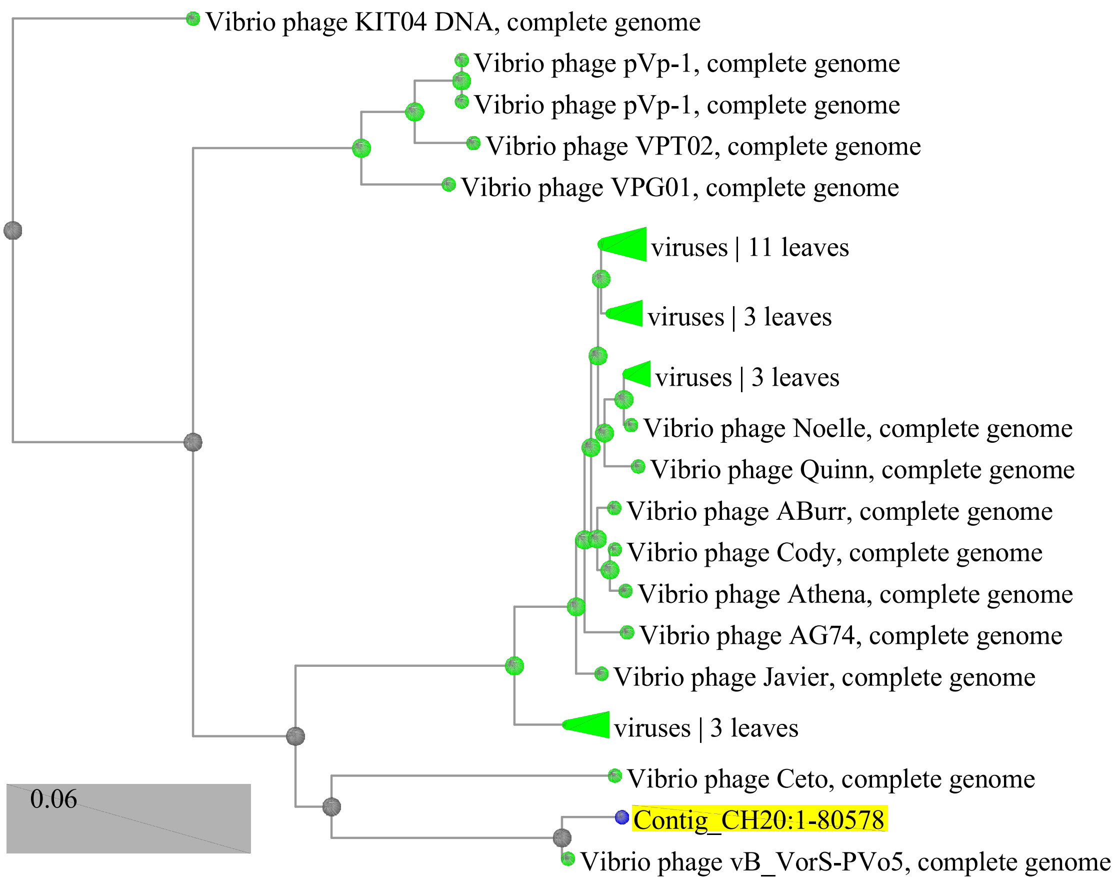

Figure S3. Tree generated by BLAST using the Fast Minimum Evolution method.

Table S1. Partial Characterization of Phage CH20.

CH20	Property
Source	mussel
Genome	DNA
Morphology	Siphoviridae
Host range	GV09
Latency	10 min
Burst size	42 virions per bacteria
